# The fractal dimension of resting state EEG increases over age in children

**DOI:** 10.1093/cercor/bhaf138

**Published:** 2025-06-12

**Authors:** Si Long Jenny Tou, Tom Chau

**Affiliations:** Institute of Biomedical Engineering, University of Toronto, Toronto, ON, Canada; Bloorview Research Institute, Holland Bloorview Kids Rehabilitation Hospital, Toronto, 150 Kilgour Rd, Toronto, ON M4G 1R8, Canada; Institute of Biomedical Engineering, University of Toronto, Toronto, ON, Canada; Bloorview Research Institute, Holland Bloorview Kids Rehabilitation Hospital, Toronto, 150 Kilgour Rd, Toronto, ON M4G 1R8, Canada

**Keywords:** brain development, children, complexity, EEG, Higuchi Fractal Dimension

## Abstract

Resting-state electroencephalography (rs-EEG) represents spontaneous neural activity and is increasingly analyzed using nonlinear measures to assess brain complexity. The Higuchi Fractal Dimension (HFD) is a widely used metric for quantifying the fractal properties of EEG signals, yet its developmental trajectory remains largely unexplored. In this study, we examined age-related changes in HFD across childhood, adolescence, and early adulthood. We analyzed eyes-closed rs-EEG from 128 channels in 83 neurotypical participants (8 to 30 yr) from the MIPDB database. To assess developmental patterns, we applied a Gaussian Linear Mixed Model with age, electrode location, and their interaction as predictors, alongside non-parametric cluster-based permutation analysis to evaluate topographical differences. We observed a significant increase in HFD with age (*P* = 0.001), most pronounced between childhood and adolescence, followed by stabilization in early adulthood. HFD also varied across electrode locations, with higher values in frontal, central, and temporal regions and lower values in parietal and occipital areas. These findings provide new insights into the maturation of neural complexity in rs-EEG, aligning with known structural and functional changes in brain development. This study contributes to the growing body of research on nonlinear EEG dynamics and their relevance to neurodevelopment.

## Introduction

Resting-state electroencephalography (rs-EEG) reflects spontaneous brain activity during wakefulness in the absence of explicit task performance. This intrinsic neural activity is considered the foundational state from which cognitive processes and behavior emerge (Newson and Thiagarajan 2019). The intricate fluctuations in rs-EEG signals are the result of a complex process comprising non-linear interactions among many neurons across different time scales and regions of the brain (Arzate-Mena 2022). Analysis of this complexity yields valuable insight into the underlying brain states of rs-EEG (Buzsaki 2006). A prevailing hypothesis posits that this complexity is crucial for the brain’s ability to adapt to both internal and external perturbations (Buzsaki 2006; Lau et al. 2022).

Fractal analysis is a means of characterizing a complex process. Fractal processes exhibit statistical self-similarity across different scales in time. The Higuchi Fractal Dimension (HFD) (Higuchi 1988) has gained widespread adoption as a metric for quantifying rs-EEG complexity, particularly due to its effectiveness in analyzing short and non-stationary time series (Kesić and Spasić 2016; Moaveninejad et al. 2025). Numerous studies have employed HFD to investigate neural complexity in various neurological disorders (Kesić and Spasić 2016), including epilepsy (Khoa et al. 2012), major depressive disorder (Kawe et al. 2019; Wu 2021), schizophrenia (Raghavendra et al. 2009), Alzheimer’s disease (Smits 2016), and autism spectrum disorder (Radhakrishnan 2021). These investigations have demonstrated HFD’s potential as both a diagnostic biomarker and a valuable feature for machine learning algorithms aimed at improving automated diagnostic tools and predictive models.

Beyond pathological conditions, brain state complexity is also modulated by aging. HFD of eyes-closed rs-EEG exhibits an inverted U-shaped relationship with age in healthy adults (20 to 89 yr), indicating an increase in brain complexity during early adulthood followed by a gradual decline in later life (Zappasodi et al. 2015).

To our knowledge, no previous study has investigated how the HFD of rs-EEG changes with age in children. It is well established in the literature that the brain undergoes extensive transformations throughout childhood and adolescence. In particular, neural development is often conceptualized as an emergent process in which localized functions become progressively integrated and distributed across broader functional networks (Smith and Thelen 2003).

From Magnetic Resonance Imaging (MRI)/functional Magnetic Resonance Imaging (fMRI) studies, several age-related patterns have been reported: a peak in gray matter volume during adolescence followed by a subsequent decrease (Blakemore 2008), an increase in white matter volume that peaks in late adulthood (Lebel and Beaulieu 2011), greater connectivity strength within functional hubs (Hwang et al. 2013) and enhanced efficiency in the default mode network (Fan 2021) from childhood to early adulthood, and an inverse U-shaped trajectory of within-network resting-state functional connectivity in cognitive control networks from childhood to adolescence to early adulthood (Sanders 2023). Meanwhile, from children to adulthood, EEG studies have documented decreases in absolute spectral density (Miskovic 2015), an upward shift in the peak of the alpha band oscillation (Miskovic 2015), a redistribution of relative spectral density toward higher frequency ranges (Miskovic 2015), and increased multiscale entropy (MSE) in fronto-central regions (Noordt and Willoughby 2021).

Despite these insights, the age-related trajectory of fractal properties (specifically, HFD) in rs-EEG remains unexplored. The present study seeks to address this gap by examining how HFD in rs-EEG varies across childhood and adolescence. Given the strong association between the complexity of the brain as a nonlinear system and cognitive function, developmental trajectories of the fractal dimension of rs-EEG may offer novel insights about brain maturation.

## Materials and methods

### Participants and data

**Fig. 1:**
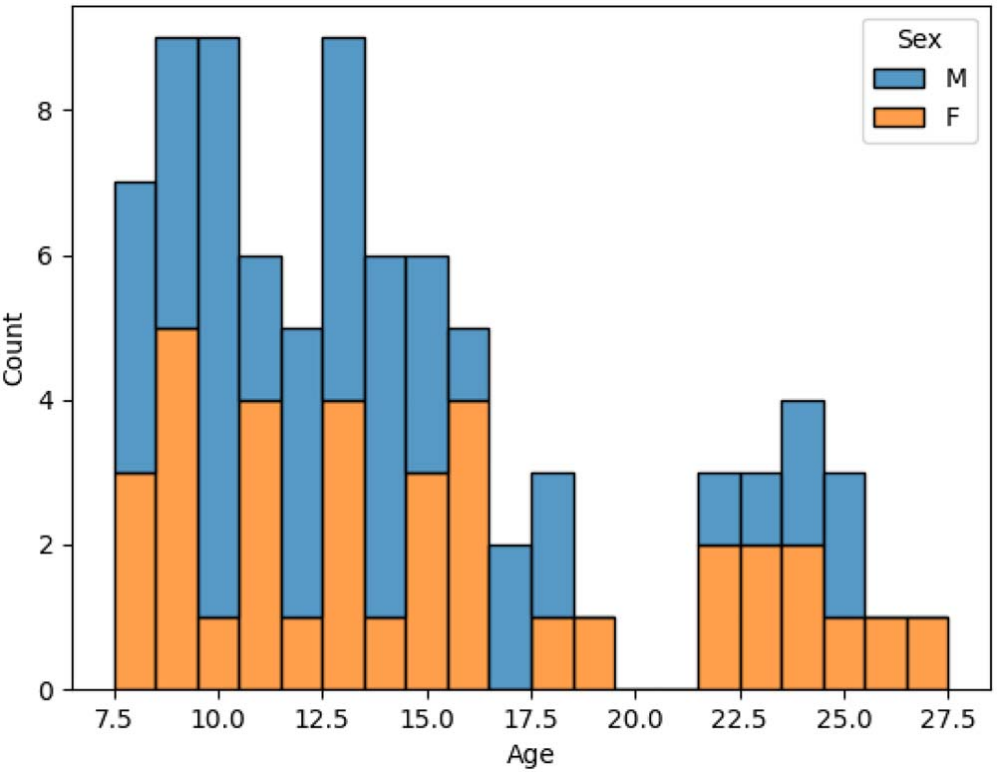
Demographics of participants included in this study.

rs-EEG data were obtained from the Multimodal Resource for Studying Information Processing in the Developing Brain (MIPDB) database, hosted by the Child Mind Institute (Langer 2017). The use of MIPDB data for this study was approved by the Research Ethics Board of Holland Bloorview Kids Rehabilitation Hospital (eREB #0517). No participants were recruited for the study. The MIPDB database includes participants aged 6 to 44 yr, with and without psychiatric and developmental diagnoses. This study included only participants aged 30 yr or younger with no reported diagnoses ($N = 83$; age 14.34 $\pm $ 5.29 yr; 46 males, 37 females). Detailed demographic information is presented in Fig. 1. Subsequent analyses grouped participants into three age cohorts: children (8 to 12 yr; 22 males, 14 females), adolescents (13 to 18 yr; 18 males, 13 females), and young adults (19 to 30 yr; 14 males, 13 females).

Five 40-s trials of eyes-closed resting-state EEG data were recorded from each participant at 500 Hz using a 128-channel Geodesic Hydrocel EEG system. Data were band-pass filtered from 0.1 to 100 Hz. Further details regarding data acquisition are available in (Langer 2017). For the current study, 20 s of each trial, starting at the fifth sec were analyzed.

### Data preprocessing

Data were preprocessed as in (Langer 2017). Briefly, noise-corrupted channels were either interpolated or removed. A notch filter (59 to 61 Hz) removed power line noise. Eye artifact correction was achieved using linear regression of electrooculogram channels, followed by principal component analysis for further noise reduction. Channels from the outermost circumference of the electrode array were excluded, resulting in a final dataset of 111 channels. For some statistical analysis, 90 of the 111 channels were categorized into 10 areas of the scalp (left and right frontal, central, temporal, parietal and occipital) following (Ravan et al. 2011).

Upon visual inspection of time-series data, spectral content, and topographical distributions of HFD values, we found substantial noise contamination in the EEG data from three participants (A00055486, 16-yr-old female; A00055296, 9-yr-old female; A00054647, 16-yr-old female). These participants were excluded from further analysis. EEG HFD was calculated from 2-s epochs of eyes-closed rs-EEG data. Each participant provided 50 such epochs.

### Higuchi Fractal Dimension

The HFD was used to quantify the complexity of rs-EEG signals, given the robustness of the method to noise (Higuchi 1988).

Let $\{x(1),x(2), \ldots , x(N)\}$ represent a rs-EEG time series of length $N$. The algorithm proceeded as follows:

1. **Time Series Segmentation:** For each embedding dimension $k = 1, 2, \dots , K_{\text{max}}$, where $K_{\text{max}}$ is a pre-defined upper limit. The time series was divided into subsequences of length $m_{k} = \lfloor N/k \rfloor $. Each subseries is constructed as:
  (1) \begin{align*}& x_{m}(k) = \left\{ x(m), x(m+k), x(m+2k), \dots, x\left(m + \left\lfloor \frac{N - m}{k} \right\rfloor k \right) \right\}\end{align*}
  where $ m $ is the starting point of the subseries, $ m = 1, 2, \dots , k $, and $ \left \lfloor \: \right \rfloor $ is the floor operator.
2. **Length Calculation:** For each subsequence $m$, the length $L_{m}(k)$ was calculated as:
  (2) \begin{align*}& L_{m}(k) = \frac{1}{k} \sum_{i=1}^{\left\lfloor \frac{N - m}{k} \right\rfloor} \left| x(m + ik) - x(m + (i - 1)k) \right| \cdot \frac{N - 1}{\left\lfloor \frac{N - m}{k} \right\rfloor k}\end{align*}
3. **Averaging the curve lengths:** For each $ k $, the mean curve length $ L(k) $ is obtained by averaging $ L_{m}(k) $ over all $ m $:
  (3) \begin{align*}& L(k) = \frac{1}{k} \sum_{m=1}^{k} L_{m}(k)\end{align*}
4. **Linear Regression:** A least-squares linear regression was performed on the log-log plot of $L(k)$ versus $k$. The slope of the resulting line represents the HFD.

To examine the influence of the $K_{\text{max}}$ parameter—recognized as a significant source of variability in HFD calculations without an established standardization in the literature (Wanliss and Wanliss 2022)—we first clarify the mathematical relationship between $k_{\text{max}}$ and the frequencies present in the data. We note that constructing each subseries $x_{m}(k)$ effectively corresponds to decimating the original time series by a factor of $k$. Consequently, higher $k_{\text{max}}$ values amplify the influence of lower-frequency components on the estimated HFD.

**Fig. 2:**
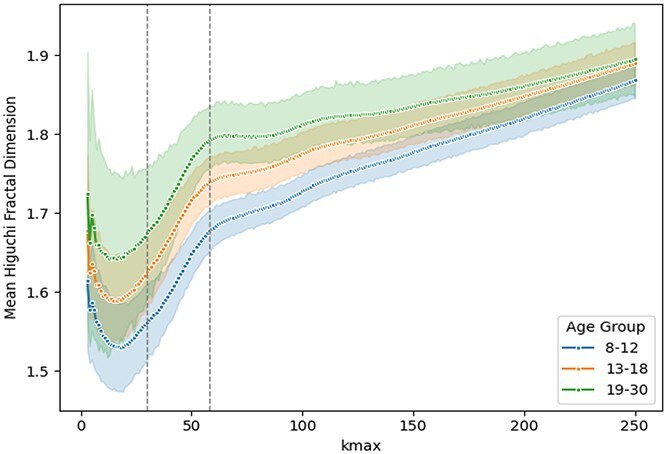
Mean HFD values across all epochs for each participant as a function of $k_{\text{max}}$, grouped by age group. Each line and its corresponding markers represent the average HFD values for participants within each age group at each $k_{\text{max}}$ value. Shaded areas indicate the 95% confidence intervals around the group means.

Considering these theoretical implications, we computed HFD across a wide range of $k_{\text{max}}$ values (from 3 to 250) using the electrode E62 in the midline(0, −6.68, 6.47), as presented in Fig. 2. From this analysis, we selected two representative values of $k_{\text{max}}$ for the main results and subsequent statistical analyses: (i) $k_{\text{max}} = 58$, corresponding to the saturation region on the HFD versus $k_{\text{max}}$ curve—an approach consistent with literature recommendations (Gómez et al. 2009; Marino 2019; Porcaro 2022; Moaveninejad et al. 2025); and (ii) $k_{\text{max}} = 30$, approximately midway between the initial value ($k_{\text{max}} = 3$) and the saturation point ($k_{\text{max}} = 58$). Using this additional, lower $k_{\text{max}}$ value allows complementary analyses that place greater emphasis on higher-frequency components.

Additionally, we computed HFD values using six distinct $K_{\text{max}}$ parameters (8, 20, 30, 50, 100, and 250). We also qualitatively replicated our main result of fractal dimension over age using Hurst exponent calculated by the Detrended Flutuation Analysis and the relationship that $FD = 2-H$ in time series. These supplementary results, detailed in the Supplementary Materials, facilitate comprehensive comparison with existing studies and reduce the risk of spurious outcomes stemming from an arbitrary choice of $K_{\text{max}}$.

### Statistical analysis

To investigate the effects of age and EEG channel location on HFD, a Generalized Linear Mixed Model (GLMM) was fitted to the data. The model was specified as:


(4) \begin{align*}& \begin{aligned} \text{HFD}_{ijklm} &= \beta_{0} + \beta_{1} (\text{Age}_{i}) + \beta_{2} (\text{electrode}{\_}\text{area}_{j}) \\ &\quad + \beta_{3} (\text{Age}_{i} \times \text{electrode}{\_}\text{area}_{j}) + \beta_{4} (\text{Sex}_{m}) + u_{k} + \epsilon_{ijklm}, \end{aligned}\end{align*}

where:

-
$ \beta _{0} $ : Intercept, representing the grand mean HFD across all electrode areas.
-
$ \beta _{1} (\text{Age}_{i}) $ : Fixed effect of age.
-
$ \beta _{2} (\text{electrode}{\_ }\text{area}_{j}) $ : Fixed effect of EEG channel location (categorical, sum-coded).
-
$ \beta _{3} (\text{Age}_{i} \times \text{electrode}{\_ }\text{area}_{j}) $ : Interaction between age and electrode area.
-
$ \beta _{4} (\text{Sex}_{m}) $ : Fixed effect to control for sex.
-
$ u_{k} \sim \mathcal{N}(0, \sigma _{u}^{2}) $ : Random effect for participant $ k $, accounting for inter-individual variability.
-
$ \epsilon _{ijklm} \sim \mathcal{N}(0, \sigma _\epsilon ^{2}) $ : Residual error.

In this model, the categorical variable electrode_area was coded using sum contrasts. Sum coding ensures that the intercept ($ \beta _{0} $) represents the grand mean of HFD across all electrode areas, and the coefficients for each electrode_area category ($ \beta _{2} $) reflect their deviations from this grand mean.

The model tested the following hypotheses:

1. **Main Effect of Age ($ \beta _{1} $)**: Whether age significantly influences HFD.
2. **Main Effect of Electrode area ($ \beta _{2} $)**: Whether HFD varies across different EEG channel locations.
3. **Interaction Effect ($ \beta _{3} $)**: Whether the effect of age on HFD depends on the electrode area.

The generalized linear mixed model (GLMM) was fitted in Python using the statsmodels package. Parameter estimates were obtained with (restricted) maximum-likelihood estimation. To control the family-wise error rate across the 19 fixed-effect tests, we applied a Bonferroni correction to the nominal $\alpha = 0.05$, yielding a significance threshold of $P < 0.0026$.

To gain deeper insights into the influence of topology and age on HFD, participants were grouped into three categories: children, adolescents, and adults. Group differences in HFD values were analyzed using a non-parametric cluster-based permutation test (Maris and Oostenveld 2007; Sassenhagen and Draschkow 2019). This approach is robust to violations of normality and homogeneity of variance assumptions, and effectively addresses the multiple comparisons problem inherent in multi-channel EEG data. The Python package mne statsṗermutation_cluster_test implementation was used. In brief, for each participant, the average HFD value was calculated for each EEG channel. A channel-wise t-statistic was then computed to compare HFD values between age groups. Channels with t-statistics exceeding a significance threshold of $\alpha /2$ (two-tailed; $\alpha = 0.05$) were considered, with adjacent channels grouped into clusters (Maris and Oostenveld 2007). A cluster-level statistic was calculated as the sum of the t-statistics within each cluster.

To evaluate the significance of the cluster-level t-statistics, a permutation test was conducted by randomly reassigning participants to age groups (10,000 permutations). For each permutation, clustering was repeated and the cluster-level statistics recomputed, yielding a null distribution of expected cluster-level statistics in the absence of age dependence. An empirically observed cluster was considered significant if its cluster-level t-statistic was more extreme than expected ($P<0.017$, $\alpha = 0.05$ with Bonferoni correction for 3 age groups) according to the null distribution. This analysis identified both significant age-related differences in average HFD and the spatial distribution of these effects across the EEG channels.

The statistical analyses, including both the GLMM and permutation clustering procedures, were repeated for $k_{\text{max}} = 58$ and $k_{\text{max}} = 30$.

## Results

We report the results obtained using both $k_{\text{max}} = 58$ and $k_{\text{max}} = 30$ together, and highlight any differences between the two.

Sex is insignificant after Bonferroni correction (*P* = 0.003) when $k_{\text{max}} = 58$, but significant (*P* = 0.002) when $k_{\text{max}} = 30$.

**Fig. 3:**
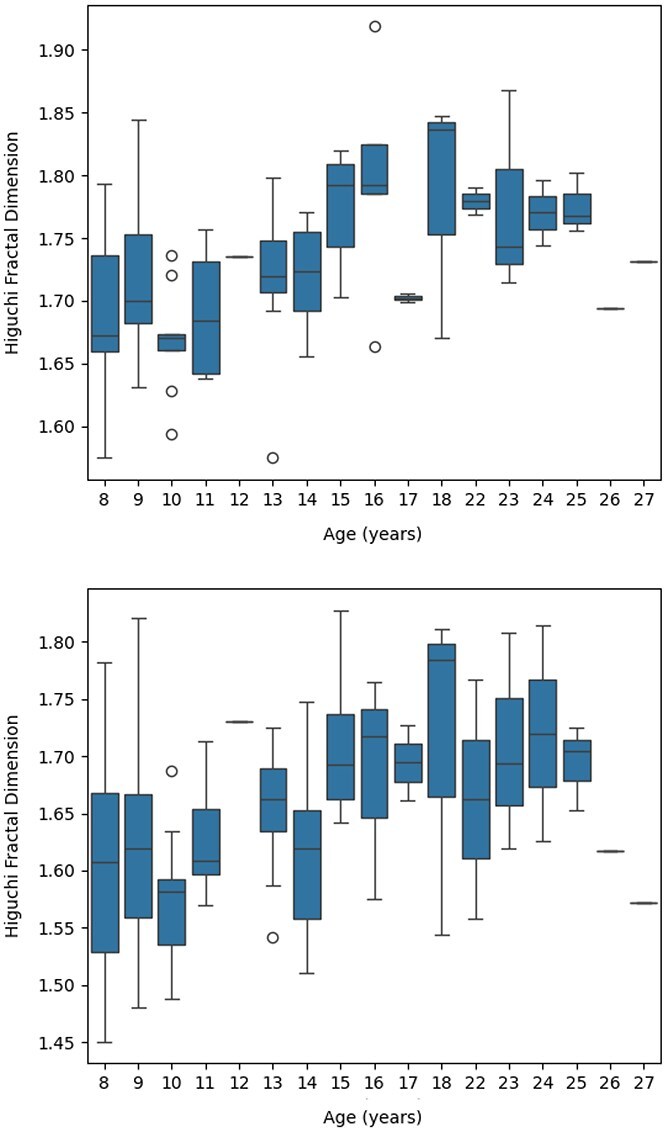
Boxplots of HFD distributions at each age group for two $k_{\text{max}}$ settings. Top: $k_{\text{max}} = 58$; bottom: $k_{\text{max}} = 30$.

The GLMM analyses yielded an intercept ($ k_{\text{max}} = 58: \beta _{0} = 1.602, P < 0.001$ ; $ k_{\text{max}} = 30: \beta _{0} = 1.500 $, $ P < 0.001 $) representing the average HFD. Age had a significant positive main effect on HFD when $k_{\text{max}} = 58$ ($ \beta _{1} = 0.005 $, $ P = 0.001 $), indicating a small global increase in HFD with age, as shown in Fig. 3. However, when $ k_{\text{max}} = 30$, the effect does not survive family-wise error correction ($ \beta _{1} = 0.004 $, $ P = 0.047 $).

Significant main effects of electrode were observed. Frontal-left (FL, $ k_{\text{max}} = 58: \beta _{2} = -0.004 $, $ P = 0.002 $; $ k_{\text{max}} = 30: \beta _{2} = 0.043 $, $ P < 0.001 $), frontal-right (FR, $ k_{\text{max}} = 58: \beta _{2} = 0.004 $, $ P = 0.007 $; $ k_{\text{max}} = 30: \beta _{2} = 0.061 $, $ P < 0.001 $), central-left (CL, $ k_{\text{max}} = 58: \beta _{2} = 0.007 $, $ P < 0.001 $; $ k_{\text{max}} = 30: \beta _{2} = 0.017 $, $ P < 0.001 $), central-right (CR, $ k_{\text{max}} = 58: \beta _{2} = 0.013 $, $ P < 0.001 $; $ k_{\text{max}} = 30: \beta _{2} = 0.032 $, $ P < 0.001 $), temporal-left (TL, $ k_{\text{max}} = 58: \beta _{2} = 0.018 $, $ P < 0.001 $; $ k_{\text{max}} = 30: \beta _{2} = 0.037 $, $ P < 0.001 $), and temporal-right (TR, $ k_{\text{max}} = 58: \beta _{2} = 0.019 $; $ k_{\text{max}} = 30: \beta _{2} = 0.026 $, implicitly determined by the coefficients of the other areas) regions showed higher HFD values compared to the average scalp HFD. Note that the frontal-left region using $k_{\text{max}} = 58$ is an exception.

In contrast, parietal-left (PL, $ k_{\text{max}} = 58: \beta _{2} = -0.011 $, $ P < 0.001 $; $ k_{\text{max}} = 30: \beta _{2} = -0.035 $, $ P < 0.001 $), parietal-right (PR, $ k_{\text{max}} = 58: \beta _{2} = -0.000 $, $ P = 0.875 $); $ k_{\text{max}} = 30: \beta _{2} = -0.029 $, $ P < 0.001 $), occipital-left (OL, $ k_{\text{max}} = 58: \beta _{2} = -0.023 $, $ P < 0.001 $; $ k_{\text{max}} = 30: \beta _{2} = -0.072 $, $ P < 0.001 $), and occipital-right (OR, $ k_{\text{max}} = 58: \beta _{2} = -0.022 $, $ P < 0.001 $; $ k_{\text{max}} = 30: \beta _{2} = -0.079 $, $ P < 0.001 $) exhibited significantly lower HFD values. Note that parietal-right coefficient using $k_{\text{max}} = 58$ is insignificant.

**Fig. 4:**
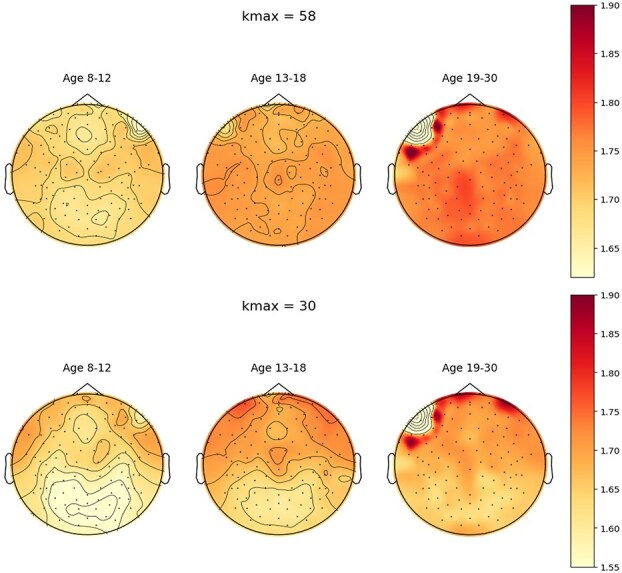
Topology of mean HFD values in children (8 to 12), adolescents (13 to 18), and adults (19 to 30) for top: $k_{\text{max}} = 58$; bottom: $k_{\text{max}} = 30$.

For visualization, Fig. 4 shows the topology of HFD in different age groups for both $k_{\text{max}} = 58$ and $k_{\text{max}} = 30$.

All interaction effects between age and electrode were significant except for PR when $k_{\text{max}} = 58$, for FL when $k_{\text{max}} = 30$, and the direction of each age $\times $ electrode coefficient was opposite to that of the corresponding main effect for electrode alone except for CL when $k_{\text{max}} = 30$. Specifically, the interaction coefficients were as follows: FL ($ k_{\text{max}} = 58: \beta _{3} = -0.0003 $, $ P < 0.001$; $ k_{\text{max}} = 30: \beta _{3} = -0.0002 $, $ P = 0.195 $, FR ($ k_{\text{max}} = 58: \beta _{3} = -0.0008 $, $ P < 0.001 $; $ k_{\text{max}} = 30: \beta _{3} = -0.0014 $, $ P < 0.001 $), CL ($ k_{\text{max}} = 58: \beta _{3} = -0.0003 $, $ P < 0.001 $; $ k_{\text{max}} = 30: \beta _{3} = 0.0005 $, $ P < 0.001 $), CR ($ k_{\text{max}} = 58: \beta _{3} = -0.0006 $, $ P < 0.001 $; $ k_{\text{max}} = 30: \beta _{3} = -0.0004 $, $ P < 0.001 $), TL ($ k_{\text{max}} = 58: \beta _{3} = -0.0007 $, $ P < 0.001 $; $ k_{\text{max}} = 30: \beta _{3} = -0.0018 $, $ P < 0.001 $), TR ($ k_{\text{max}} = 58: \beta _{3} = -0.001 $; $ k_{\text{max}} = 30: \beta _{3} = -0.0011 $, implicitly calculated), PL ($ k_{\text{max}} = 58: \beta _{3} = 0.0009 $, $ P < 0.001 $; $ k_{\text{max}} = 30: \beta _{3} = 0.0009 $, $ P < 0.001 $), PR ($ k_{\text{max}} = 58: \beta _{3}= 0.0002 $, $P = 0.082$; $ k_{\text{max}} = 30: \beta _{3}= 0.0006 $, $ P < 0.001 $), OL ($ k_{\text{max}} = 58: \beta _{3} = 0.0014 $, $ P < 0.001 $; $ k_{\text{max}} = 30: \beta _{3} = 0.0014 $, $ P < 0.001 $), and OR ($ k_{\text{max}} = 58: \beta _{3} = 0.0012 $, $ P < 0.001 $; $ k_{\text{max}} = 30: \beta _{3}= 0.0016 $, $ P < 0.001 $). These results indicate that when controlling for age, the topological differences in HFD across electrodes were less pronounced compared to the unadjusted main effects. Collectively, these findings demonstrate that HFD is significantly influenced by age and electrode location.

**Fig. 5:**
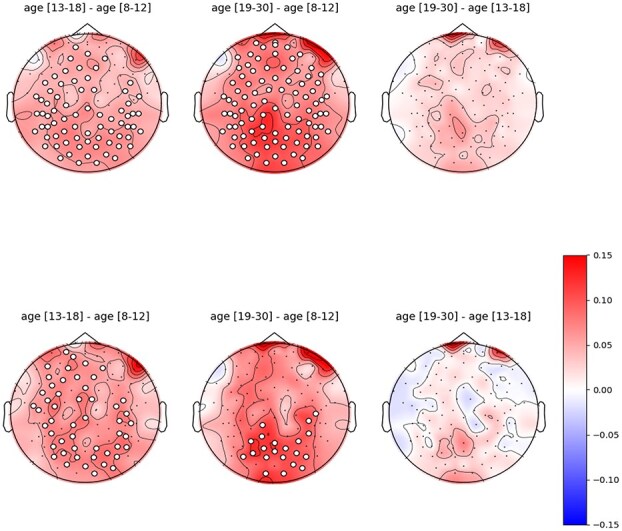
Topology of mean differences in HFD values between age groups. Left: children vs. adolescents; center: children vs. adults; right: adolescents vs. adults. Red signifies an increase in mean HFD while blue denotes a decrease in mean HFD. The white circles represent electrodes belonging to significant clusters. Top: $k_{\text{max}} = 58$; bottom: $k_{\text{max}} = 30$. The color scale of the top and bottom figures is the same.

The GLMM results were corroborated by the findings from permutation clustering, which indicated that HFD values are significantly influenced by age across a wide array of electrodes. Under both $k_{\text{max}} = 58$ and $k_{\text{max}} = 30$ settings, a large cluster of electrodes covering a substantial portion of the scalp was found to be significant when comparing children and adolescents ($k_{\text{max}} = 58: P = 0.0014$; $k_{\text{max}} = 30: P = 0.029$). In the comparison between children and adults, a significant cluster was identified covering a substantial portion of the scalp under $k_{\text{max}} = 58$ ($P < 0.001$), but under $k_{\text{max}} = 30$, only in the posterior region of the scalp (*P* = 0.011). No significant clusters were observed when comparing adolescents and adults in either $k_{\text{max}}$ settings. These results are shown in Fig. 5.

These results suggest that the primary increase in HFD occurs during the transition from childhood to adolescence, and that HFD stabilizes before adulthood.

## Discussion

Our finding of escalating HFD with age in rs-EEG of children under $k_{\text{max}} = 58$ is consistent with previous studies reporting developmental increases in EEG complexity, such as that reflected in growth of MSE (Noordt and Willoughby 2021) and maturational changes in brain network-based metrics such as higher average clustering, longer path length, and reduced weight dispersion (Boersma 2011). The age effect was weakened and did not survive family-wise error correction when $k_{\text{max}} = 30$, suggesting that age influences the full frequency spectrum of the signal rather than being confined to higher-frequency components.

Additional insights into age-related patterns in HFD can be drawn from neuroimaging studies of white matter. In Fig. 5, we observed a significant increase in HFD from childhood to adolescence, but no notable differences between adolescents and adults. This finding parallels studies indicating that white matter development is substantial in childhood and early adolescence, with most changes complete by late adolescence, particularly in the projection and commissural tracts (Lebel and Beaulieu 2011).

Literature on adult data further suggest a possible connection between white matter connectivity and EEG complexity. In adults, the fractal dimension of the hemispheric white matter tracts exhibits a U-shaped pattern across the lifespan, peaking around 50 yr of age in the left hemisphere and around 40 yr of age in the right (Farahibozorg et al. 2015). Interestingly, this U-shaped pattern and the timing of its peak also appear in the fractal dimension of rs-EEG with aging (Zappasodi et al. 2015; Smits 2016).

From a topographical perspective, our results under both $k_{\text{max}}$ settings indicate that HFD increases in most scalp regions from childhood to adolescence (Fig. 5). However, from childhood to adulthood, no significant differences were found in the anterior regions when using $k_{\text{max}} = 30$.

In addition, in the GLMM analysis, the magnitude of the coefficients for the effect of electrode position ($\beta _{2}$) is smaller when using $k_{\text{max}} = 58$ compared to $k_{\text{max}} = 30$. This suggests that smaller values of $k_{\text{max}}$ may be more sensitive for detecting topological changes in HFD associated with development. Visual inspection of Fig. 4 further supports this observation, indicating that developmental differences in topological features may reside primarily in higher-frequency components.

Topological difference during development may be understood in the context of resting-state functional connectivity (rsFC) from MRI BOLD data. Specifically, within-network rsFC in cognitive control networks increases from childhood to early adolescence, but from middle to late adolescence, within-network rsFC in several association networks decreases (Sanders 2023). This late adolescent decrease in rsFC may explain the plateau in HFD observed in our data when $k_{\text{max}} = 30$.

Furthermore, research on brain development indicates that maturation begins in sensorimotor regions and progressively extends to the dorsal and parietal areas, the superior temporal regions, and the dorsolateral prefrontal cortex in later childhood and adolescence (Marsh et al. 2008). The topographical patterns of the mean HFD development shown in Fig. 4 broadly align with this progression, and is particularly evident under both $k_{\text{max}} = 58$ and $k_{\text{max}} = 30$ setting.

Finally, it should be noted that the relative power across the EEG frequency bands is theoretically related to HFD through the spectral scaling decay rate ($\beta $, the slope of the logarithmic power spectrum). Specifically, $FD = \text{Euclidean dimension} \;+\; \frac{(3 - \beta )}{2}$. Previous research has reported a redistribution of the relative spectral density toward higher frequencies as children age (Gasser et al. 1988; Miskovic 2015; Markovska-Simoska and Pop-Jordanova 2017), which corresponds to a decrease in $\beta $ and thus supports our finding of an increasing HFD.

## Supplementary Material

supplementary_bhaf138
