# Supplementary material for: The fractal dimension of resting state EEG increases over age in children
Source: Cereb Cortex. 2025 Jun 12;35(6):bhaf138. doi: 10.1093/cercor/bhaf138 (PMC12159291; doi:10.1093/cercor/bhaf138)
Supplement: supplementary_bhaf138 [file supplementary_bhaf138.pdf]

Supplementary Materials

The Fractal Dimension of Resting-State EEG Increases over Age in Children

Si Long Jenny Tou[1,2,*] and Tom Chau[1,2]

[1]Institute of Biomedical Engineering, University of Toronto, Toronto, ON, Canada
[2]Bloorview Research Institute, Holland Bloorview Kids Rehabilitation Hospital, Toronto, ON, Canada

[*]Corresponding author: sl.tou@mail.utoronto.ca
150 Kilgour Rd, Toronto ON M4G 1R8, Canada

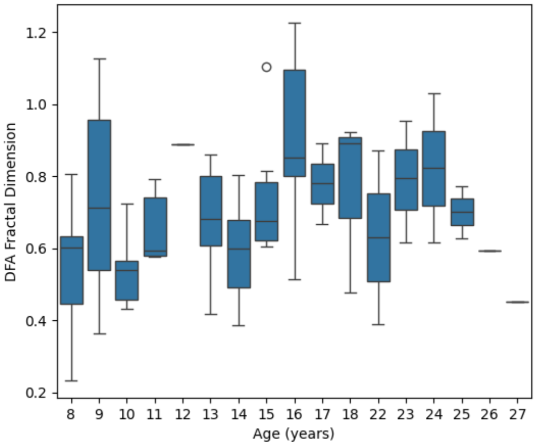

Figure A1: Boxplots showing the distribution of fractal dimension (FD) values, calculated using detrended fluctuation analysis (DFA), across age groups. This figure provides a reference baseline for comparison with HFD results, helping to mitigate potential spurious effects associated with the choice of the k_{max} parameter in HFD estimation.

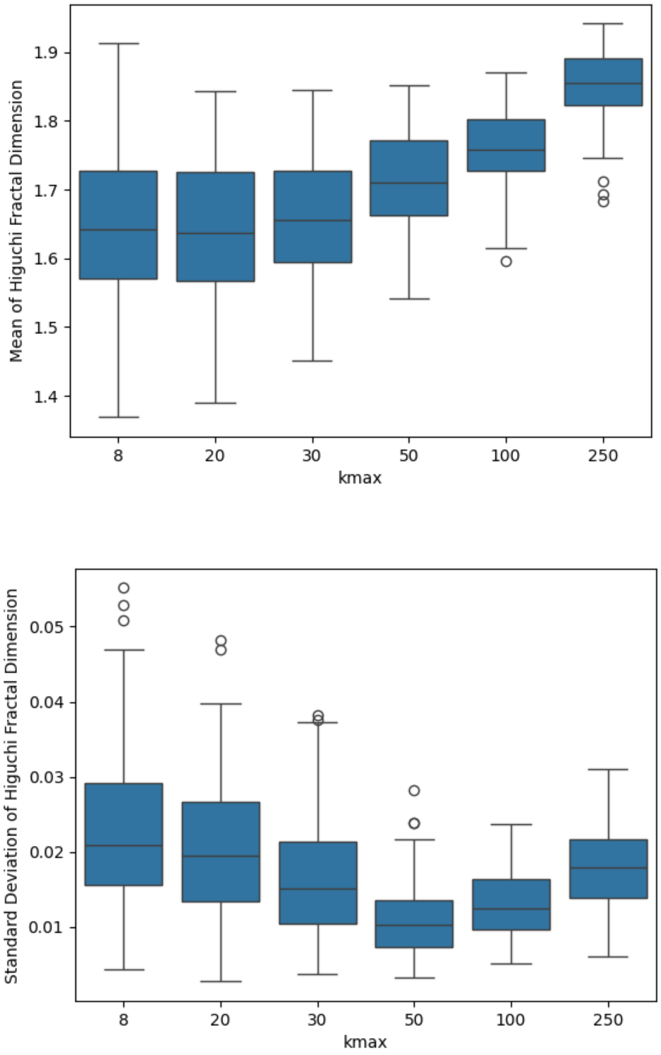

Figure A2: The mean and standard deviation of HFD values across all participants and all channels.

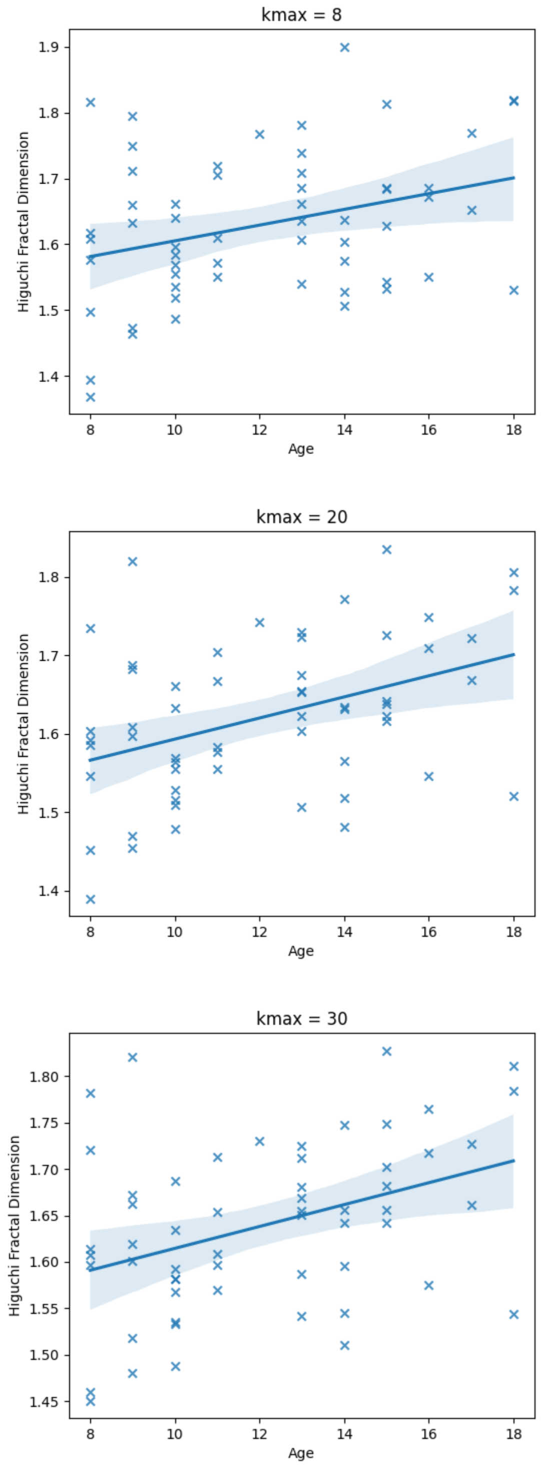
kmax = 8
Higuchi Fractal Dimension
Age
kmax = 20
Higuchi Fractal Dimension
Age
kmax = 30
Higuchi Fractal Dimension
Age

Figure A3:

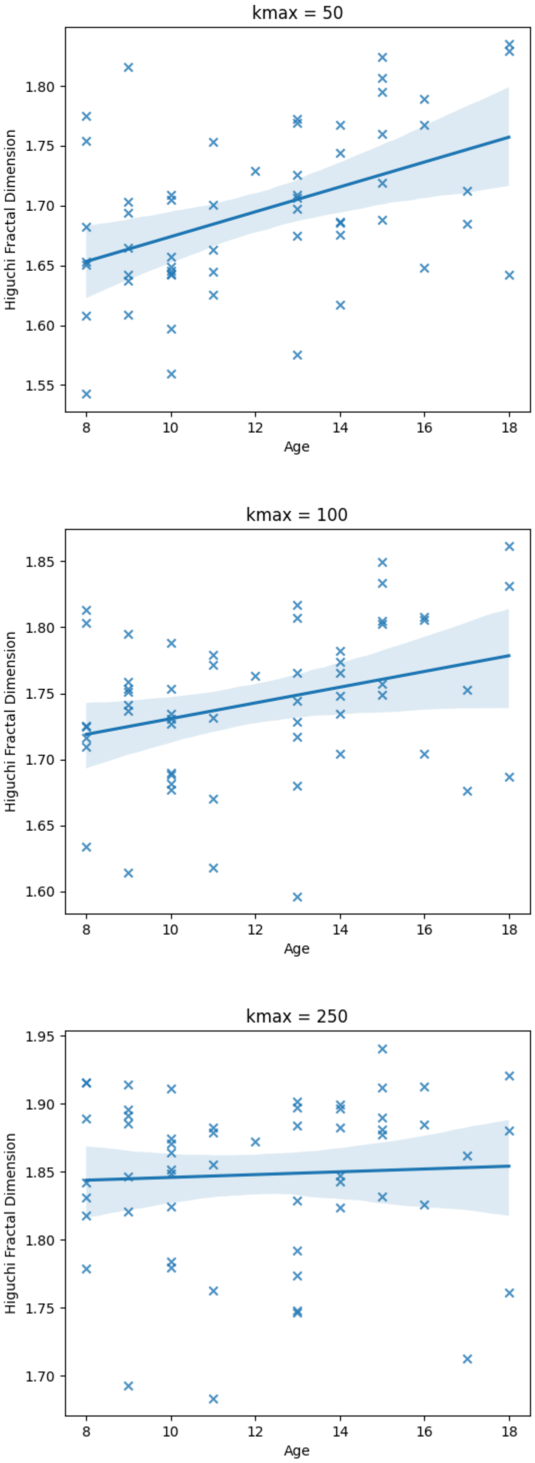

Figure A4: HFD values over age for various k_{max} values.

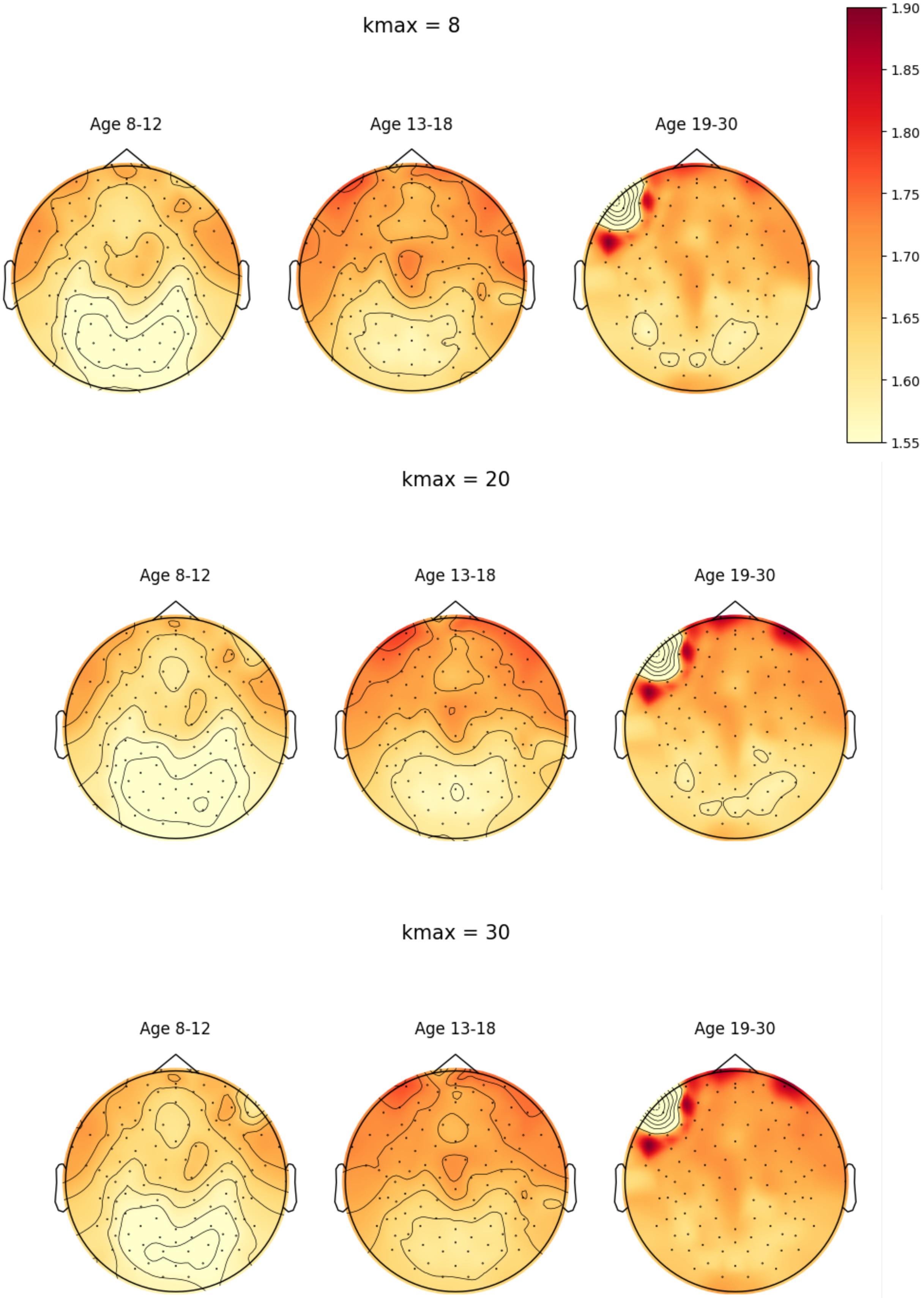
kmax = 8
Age 8-12
Age 13-18
Age 19-30
1.90
1.85
1.80
1.75
1.70
1.65
1.60
1.55
kmax = 20
Age 8-12
Age 13-18
Age 19-30
kmax = 30
Age 8-12
Age 13-18
Age 19-30

Figure A5:

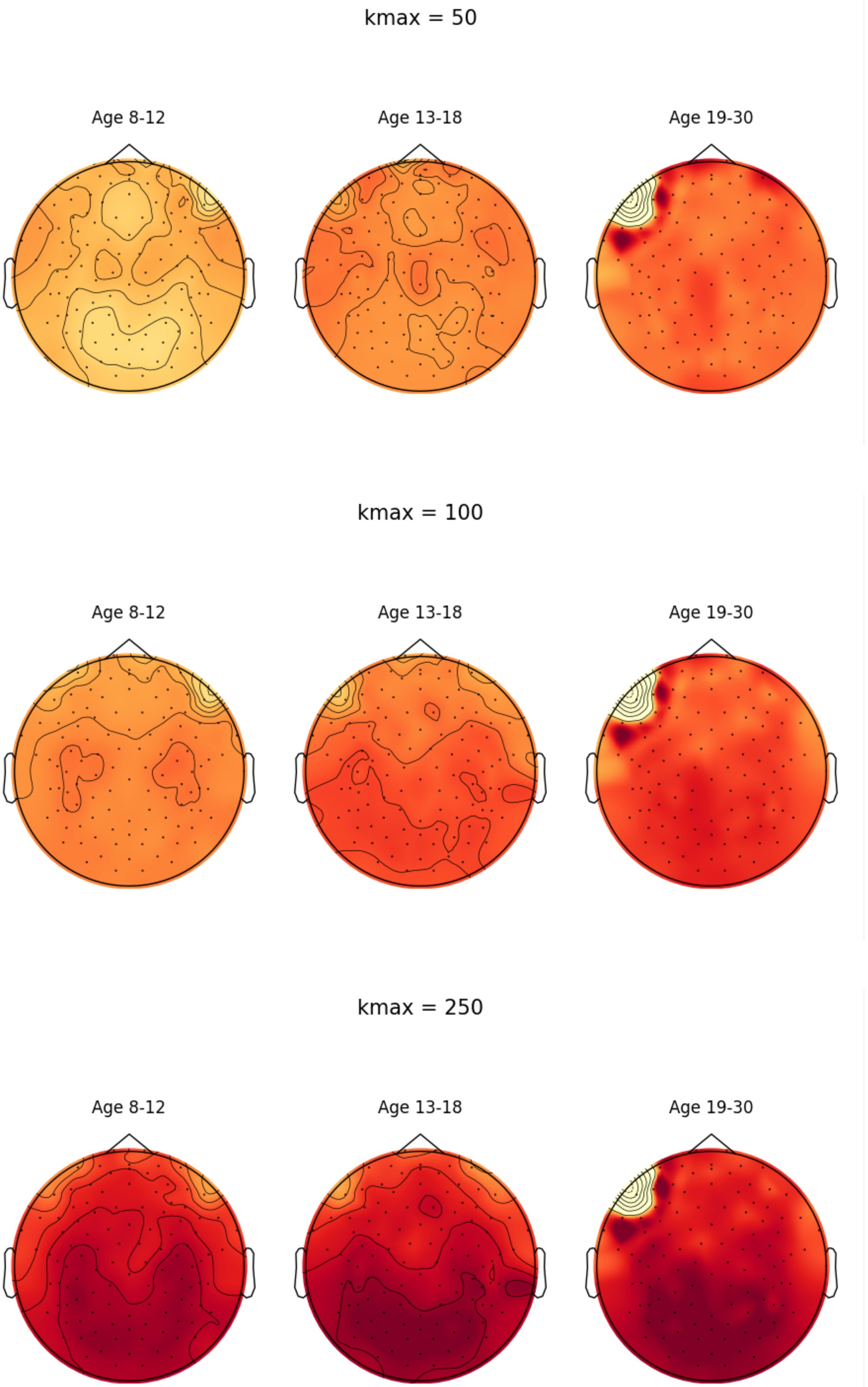

Figure A6: Topological relationships in the Higuchi Fractal Dimension (HFD) across different age groups. The figure shows that consistent topological relationships are observed up to $K_{max} = 30$; beyond this value, the relationships break down.
